# Lack variation of low slow‐wave activity over time in the frontal region in NREM sleep may be associated with dyskinesia in Parkinson's disease

**DOI:** 10.1111/cns.70058

**Published:** 2024-10-07

**Authors:** Yi‐Ming Wang, Jun‐Yi Liu, Fan Gao, Wei‐ye Xie, Jing Chen, Han‐Ying Gu, Fen Wang, Chong‐Ke Zhong, Kai Li, Sheng Zhuang, Xiao‐Yu Cheng, Hong Jin, Jin‐Ru Zhang, Cheng‐Jie Mao, Chun‐Feng Liu

**Affiliations:** ^1^ Department of Neurology and Clinical Research Center of Neurological Disease The Second Affiliated Hospital of Soochow University Suzhou China; ^2^ Department of Neurology Du Shu Lake Hospital Affiliated to Soochow University Suzhou China; ^3^ Jiangsu Key Laboratory of Neuropsychiatric Diseases and Institute of Neuroscience Soochow University Suzhou China; ^4^ Department of Epidemiology, School of Public Health Medical College of Soochow University Suzhou China

**Keywords:** dyskinesia, EEG spectral density, parkinson's disease, polysomnography, slow wave activity

## Abstract

**Objective:**

Levodopa‐induced dyskinesia (DYS) adversely affects the quality of life of Parkinson's disease (PD) patients. However, few studies have focused on the relationship between DYS and sleep and electroencephalography (EEG). Our study aimed to establish the objective physiological indicators assessed by polysomnography (PSG) that are associated with DYS in PD patients.

**Method**s**:**

We enrolled 122 patients with PD, divided into two groups: PD with DYS (*n* = 27) and PD without DYS group (non‐DYS, *n* = 95). The demographics and clinical characteristics and sleep assessment in the two groups were collected. More importantly, overnight six‐channel PSG parameters were compared in the two groups. We also compared different bands and brain regions of average power spectral density within each group.

**Results:**

Compared with the non‐DYS group, the DYS group tended to have a significantly higher percentage of nonrapid eye movement sleep (NREM). Gender, levodopa equivalent daily dose (LEDD), rapid eye movement (REM) sleep (min), and the NREM percentage were positively correlated with the occurrence of DYS. After adjusting for gender, disease duration, LEDD, taking amantadine or not, and Montreal Cognitive Assessment (MoCA), NREM%, N3%, and REM (min), the percentage of NREM sleep (*p* = 0.035), female (*p* = 0.002), and LEDD (*p* = 0.005), and REM sleep time (min) (*p* = 0.012) were still associated with DYS. There was no significant difference in whole‐night different bands of average power spectral density between two groups. There was no significant difference in normalized average power spectral density of slow wave activity (SWA) (0.5–2 Hz, 0.5–4 Hz, and 2–4 Hz) of early and late NREM sleep in the DYS group. Dynamic normalized average power spectral density of SWA of low‐frequency (0.5–2 Hz) reduction in the frontal region (*p* = 0.013) was associated with DYS in logistic regression after adjusting for confounding factors.

**Conclusion:**

PD patients with DYS have substantial sleep structure variations. Higher NREM percentage and less REM percentage were observed in PD patients with DYS. Dynamic normalized average power spectral density of low‐frequency (0.5–2 Hz) SWA reduction in the frontal area could be a new electrophysiological marker of DYS in PD.

## Graphical abstract

PD patients with dyskinesia have substantial sleep structure variations. A higher NREM ratio and a lower REM ratio were observed in PD patients with dyskinesia. Normalized average power spectral density of low‐frequency (0.5–2 Hz) SWA reduction in the frontal area could be a new electrophysiological marker of dyskinesia in PD.

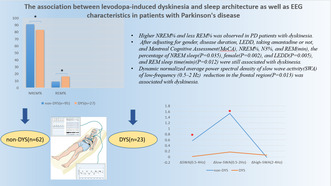

## 1 INTRODUCTION

Parkinson's disease (PD) is a neurodegenerative disorder mainly characterized by insufficient dopamine in the substantia nigra–striatum system.^1^ Levodopa is currently recognized as the most effective drug for the symptomatic treatment of PD, but with the progression of PD, most patients suffer from motor complications after long‐term dopaminergic treatment.^2^ Levodopa‐induced dyskinesia (LID) is a common motor complication. Studies have shown that PD patients who had more than 10 years of levodopa treatment had an approximately 89% ratio of dyskinesia (DYS).^3^

The mechanism of DYS is not fully understood. The pathophysiology of DYS involves serotonergic and GABAergic systems in addition to degeneration of the substantia nigra striata system and high‐dose levodopa administration. Also, the duration of levodopa administration may be relevant. The occurrence of LID is closely related to the formation of pathological long‐term potentiation (LTP) in cortical striatum induced by long‐term treatment with levodopa. An animal experimental study shows that the occurrence and development of LID is closely related to the enhancement of synaptic activity in the cortical striatum.^4^ For human beings, degeneration of dopamine neurons in the cerebral cortex can lead to abnormal plasticity of the cerebral cortex. Studies have shown that PD patients with DYS tend to have worse cerebral cortical plasticity.^5^, ^6^ It is known that slow wave activity (SWA) is associated with cortical plasticity. SWA in nonrapid eye movement (NREM) sleep is due to a progressive decrease in the strength of cortical connections, and there is recent compelling evidence associated SWA with cortical synaptic strength and therefore measuring the reduction in SWA from early (the first 1 h at the beginning of the light period) to late (the last 1 h of the light period) NREM sleep has often been used as a direct measure of synaptic homeostasis hypothesis.^7^, ^8^ Changes in cortical SWA in DYS have been observed in animal models.^9^ Our previous study demonstrated that Pittsburgh Sleep Quality Index (PSQI) score is associated with DYS in PD. Subjective sleep quality and sleep latency are associated with DYS in PD patients.^10^ These findings suggest that there may be some association between sleep and DYS in PD patients.

Sleep disorder is one of the common non‐motor symptoms in PD patients. The mechanism behind sleep disorders in PD patients is multifactorial and complicated including degeneration of thalamocortical pathway, brainstem nuclei, including the glutamatergic peri‐locus coeruleus combined with abnormalities in brainstem locomotor centers.^11^, ^12^ The dopaminergic system also plays an important role in the regulation of the sleep–wake cycle.^13^ In addition, drugs, other nonmotor symptoms, and poor control of nocturnal motor symptoms affect sleep. Sleep deprivation impacts structural plasticity and synaptic strength.^14^ Slow‐wave sleep affects synucleinopathy and regulates proteostatic processes in mouse models of PD.^15^ The close relationship between slow‐wave sleep and nonmotor symptoms of PD has gained increasing attention in recent years. Relevant studies have shown that slow‐wave sleep and electroencephalography (EEG) delta spectral power are associated with cognitive function in PD.^16^ So far, there has been a clinical study focusing on the association between LID and slow‐wave sleep in PD patients.^17^ By video polysomnography (PSG)‐high‐density EEG, this well‐designed study suggested that DYS patients do not have adequate synaptic downscaling during sleep.^17^ The promising results of this study provide a unique framework.

So far, some studies have partly clarified the relationship between DYS and slow‐wave sleep,^9^, ^17^ but the specific mechanism between DYS and slow‐wave sleep remains unclear. To broaden the view of previous studies, confirm the relationship between sleep and DYS objectively, and investigate new electrophysiological markers of DYS in PD patients, we further expanded the sample size, explored the characteristics of sleep structure and EEG in PD patients with DYS by performing six‐channel (F3/4, C3/4, O1/2) PSG.

## 2 METHODS

### 2.1 Patients

**FIGURE 1:**
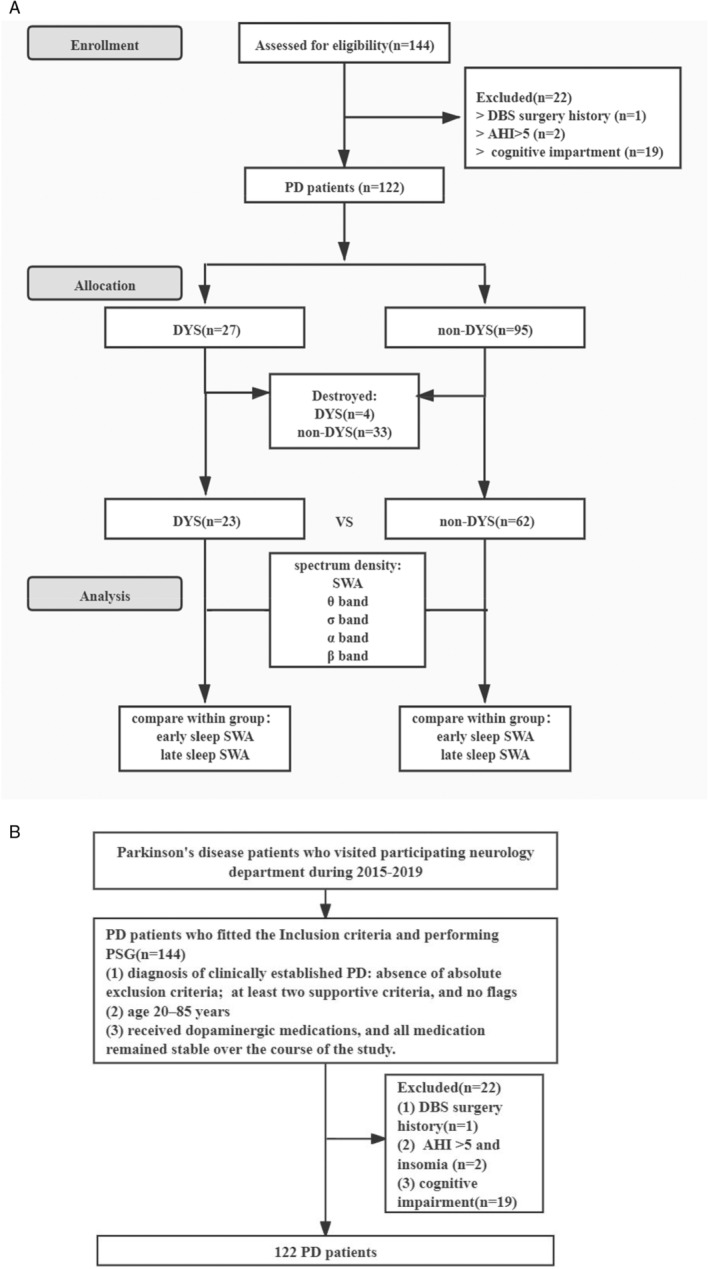
(A) Schematic flow chart of the study. (B) Flow chart of patient selection. AHI, Apnea–Hypopnea Index; DBS, deep brain stimulation; DYS, dyskinesia; NREM, nonrapid eye movement; PD, Parkinson's disease; SWA, slow‐wave activity.

We enrolled 144 PD patients at the Department of Neurology at the Second Affiliated Hospital of Soochow University from March 2015 to November 2019. Inclusion criteria included: (1) diagnosis of clinically established PD: absence of absolute exclusion criteria; at least two supportive criteria, and no flags^18^; (2) age 20–85 years; and (3) all patients received dopaminergic medications, and all medication remained stable over the course of the study. Exclusion criteria included: (1) secondary parkinsonism syndrome, atypical parkinsonian syndrome, Alzheimer's disease and epilepsy; (2) patients who had experienced deep brain stimulation surgery; (3) Apnea–Hypopnea Index (AHI) >5^17^ and insomnia; and (4) cognitive impairment, as defined by a Mini‐Mental State Examination (MMSE) <24. Finally, 122 PD patients were included for further analysis. All participants signed an informed consent form (Figure 1B).

DYS was assessed using the Unified Parkinson's Disease Rating Scale (UPDRS) Part IV based on item 32. In our study, we only use the DYS items for the correlation analysis. Patients were divided into two groups: PD with DYS (*n* = 27) and PD without DYS (non‐DYS, *n* = 95). As the EEG data of some patients were damaged during the equipment update iteration in our center, we were unable to obtain the PSG EEG data of these patients. As a result, 85 patients who had complete EEG data were divided into two groups: non‐DYS (*n* = 62) and DYS (*n* = 23). The EEG characteristics of 85 patients were analyzed by spectral analysis.

This study was approved by the Ethics Committee of the Second Affiliated Hospital of Soochow University and informed consent was obtained from all participants. All participants provided written informed consent to participate in the study.

### 2.2 Study design

#### 2.2.1 Clinical data

Baseline demographic data, including age, gender, disease duration, and medication information, were collected. Calculation of a levodopa equivalent daily dose (LEDD) for each patient was based on theoretical equivalence to levodopa as follows: levodopa dose + levodopa dose × 1/3 if on entacapone +piribedil (mg) + pramipexole (mg) × 100 + selegiline (mg) × 10 + amantadine (mg) + controlled release levodopa (mg) × 0.75.^19^

UPDRS scale and Hoehn & Yahr (H‐Y) stage were applied to assess the motor symptoms during the medication “on” state. PSQI were completed to evaluate the sleep quality in these patients in recent months.^20^, ^21^ Hamilton Anxiety Scale (HAMA),^22^ Hamilton Rating Scale for Depression (HRSD),^23^ and MMSE scale,^24^ Montreal Cognitive Assessment Scale (MOCA)^25^ were applied to evaluate the emotion and cognition respectively. We reported rapid‐eye‐movement behavior disorders (RBD) or not of each patient. RBD was diagnosed based on the *International Classification of Sleep Disorders‐third edition* (ISCD‐3).^26^

#### 2.2.2 PSG

Standard overnight sleep monitoring was recorded for each subject in a sleep center using an E‐Series System digital recorder manufactured by Compumedics, Australia. All polysomnographic recordings included six EEG channels: frontal lobe (F3, F4), central lobe (C3, C4), occipital lobe (O1, O2), and two reference electrodes placed on the bilateral mastoid (A1, A2). The PSG also had channels with the ability to record ECG, EMG of anterior tibial muscle surfaces for recording PLMS, along with the measurement of oronasal flow and pulse oximetry. All data were collected by senior technicians in accordance with the American Academy of Sleep Medicine (AASM) standards to perform sleep staging on the PSG data of the study subjects.^27^ Sleep parameters included: total sleep time (TST), sleep efficiency (SE), sleep latency (SL), wakefulness or arousal duration, REM sleep and nonrapid eye movement (NREM) sleep, and the percentage of each sleep pattern was recorded. NREM sleep was divided into N1, N2, and N3. N3 is defined as the deepest stage of NREM sleep. The NREM% is calculated by (N1 + N2 + N3)/total sleep time. According to the sleep record, the presence of sleep‐related apnea and the AHI were recorded by a senior technician. Electrical activity from frontal, central, and occipital brain regions, eye movements, and chin EMG are all used to determine the sleep and wake stages of a senior technician. Insomniacs who have difficulty entering into the sleep stage will be excluded.

### 2.3 Data analysis

#### 2.3.1 EEG analysis

PSG data were exported in European data format (EDF). We performed spectral analysis from six EEG derivations covering frontal, central, and occipital regions (F3, F4, C3, C4, O1, and O2). We analyzed the data further using EEGLAB and custom‐made MATLAB codes. EEG data were sampled at 256 Hz. Preprocessing of EEG and spectral analysis were performed filtering (0.3 Hz high pass and 35 Hz low‐pass). The EEG data took 30 s as a frame. After the EEG data were pre‐processed, the NREM sleep data were manually extracted according to the written stages of senior technicians with reference to AASM standard markers,^27^ and the periods containing artifacts related to arousal and PLMS due to leg movement were excluded. NREM sleep data were extracted during N2 and N3.^28^ According to Amato's study, epochs containing arousal and body movement were excluded, and the remaining data were subdivided into 10 equal segments.^17^ We defined the first 5 parts as early sleep, and the last 5 parts as late sleep because we found that N3 was expressed from the first segment. The Welch method calculates power across several overlapped segments via a Hamming‐windowed fast Fourier transform (FFT).^29^ Specifically, for each epoch, average power spectral density was calculated over a series of windows 6 s in length with 4 s overlap. The whole‐region EEG power spectral density was averaged across six channels. The EEG power spectral density of the frontal region was averaged across F3/4. Central region and occipital region power spectral densities were averaged across C3/C4 and O1/O2 and we compared static EEG average power spectral density of different bands of all xxNREM sleep between two groups. EEG average power spectral density in the NREM sleep of two groups was compared for the following frequency bands: SWA (0.5–4 Hz), *θ* band (4–8 Hz), α band (8–13 Hz), *σ* band (13–16 Hz), and *β* band (16–30 Hz). These whole‐night band powers of N2 and N3 were normalized to a total of 0.5–30 Hz power. The normalized average power spectral density was calculated by dividing the absolute power within a specific frequency band by the total power of 0.5–30 Hz.^30^ Meanwhile, the average power spectral density of early or late sleep was separately calculated for the 0.5–4 Hz, low (0.5–2 Hz), and high (2–4 Hz) δ frequency bands. We compared early and late static SWA (0.5–4 Hz, 0.5–2 Hz, 2–4 Hz) within each group. Individual early and late absolute EEG power spectral density was normalized using the same time frame. For example, early or late SWA (0.5–4 Hz, 0.5–2 Hz, 2–4 Hz) was normalized to a total of 0.5–30 Hz power spectral density during early or late N2 and N3. We reported normalized average power spectral density of early or late EEG SWA. In addition, we also calculated dynamic normalized average power spectral density SWA (0.5–4 Hz, 0.5–2 Hz, and 2–4 Hz) of each group expressed as ΔSWA, and the calculation method was: normalized average power spectral density of ΔSWA = normalized average power spectral density of early sleep SWA‐normalized average power spectral density of late sleep SWA. The research flow chart is shown in Figure 1.

#### 2.3.2 Statistical analysis

Statistical analysis was conducted using SPSS version 23 (IBM, Armonk). Continuous variables were expressed as mean ± standard deviation (SD) or median (interquartile range [IQR]) and were compared using Student's *t*‐test or Kruskal–Wallis test. Testing data normality was used for Kolmogorov–Smirnov. Categorical variables were expressed as frequency (%) and were compared using the chi‐square test. For within‐group analyses, the two‐sample *t*‐test was used for data conforming to normal distribution, and the Wilcoxon‐signed rank test was used for data not conforming to normal distribution. For between‐group analyses, independent t‐test were used for continuous variables following a normal distribution. A binary logistic regression model was used to assess the association between sleep parameters and EEG parameters with DYS in PD patients. Each independent variable was analyzed by univariate analysis, and variables with *p* < 0.2 were then entered into a multivariate analysis as a confounding factor. Variables that have a clear correlation with DYS in the previous study are also included as confounding factors.^10^, ^16^, ^28^, ^31^ Odds ratios (ORs) and 95% confidence intervals (95% CIs) were used to evaluate the risk of DYS after adjusting for age, gender, age at onset of PD, LEDD, disease duration, taking amantadine or not, MoCA scores, N3%, REM sleep time (min). All *p* values were two‐tailed, and a significance level of 0.05 was used.

## 3 RESULTS

### 3.1 Demographics of different groups of PD patients

The baseline characteristics are presented in Table 1. There were 73 male patients (59.8%) and 49 female patients (40.2%).

**TABLE 1:** Baseline characteristics in PD patients.

	DYS (*n* = 27)	Non‐DYS (*n* = 95)	*p* value
Male sex	11 (40.7%)	62 (65.3%)	**0.022**
Age, years	66 (61–70)	65 (60–70)	0.493
Age at onset, years	57 (50.17–64)	61 (54–67)	0.138
Duration of disease, month	96 (48–132)	48 (12–84)	**<0.001**
LEDD, mg	750 (600–1050)	350 (225–575)	**<0.001**
H‐Y stage	3 (2–3)	2 (1.5–2.5)	**0.003**
Taking amantadine	11 (39.3%)	17 (60.7%)	**0.013**
UPDRS I	4 (2–6)	3 (1–5)	0.098
UPDRS II	15 (10–19)	10 (7–14)	**0.007**
UPDRS III	26.54 ± 12.86	24.74 ± 12.55	0.537
UPDRS IV	5.5 (3.75–7.25)	2 (1–4)	**<0.001**
PSQI	11.5 (3–15.25)	7 (5–10)	0.179
HRSD	14.5 (4.5–18.5)	8 (4–18)	0.277
HAMA	11 (2.75–16)	7 (4–11.5)	0.348
MMSE	27 (24–28)	28 (25–29)	0.117
MoCA	23 (20–26)	23 (19–25)	0.95
RBD	12 (44.4%)	36 (37.9%)	0.656
TST, min	328 (289–433)	375 (293–440)	0.622
Sleep efficiency, %	59.94 ± 14.05	65.35 ± 18.05	0.153
Sleep latency, min	3.5 (1.0–17.5)	8.5 (1.5–21.5)	0.218
Wakefulness or arousal duration, min	22.78 ± 10.15	25.11 ± 11.62	0.348
N 1, min	41 (19.5–63.5)	34 (16–62)	0.568
N 1, %	16.9 (6–22.5)	10 (5.2–21.5)	0.355
N 2, min	182.17 ± 74.55	177.44 ± 74.04	0.771
N 2, %	55.97 ± 18.91	49.60 ± 14.45	0.114
N 3, min	53.96 (17.59–67.47)	58.44 (34.48–93.28)	0.185
N 3, %	13.70 (4.8–22.5)	16.50 (9.7–28.9)	0.228
REM, min	35 (13.5–54)	62 (31–83.5)	**0.006**
REM, %	8.9 (3.8–18.7)	16.4 (10.3–21.8)	**0.01**
REM latency, min	205.5 (110.5–285.5)	131.5 (75–199)	0.055
NREM, %	91.1 (82.05–93.4)	83 (78–88.4)	**0.006**
NREM, min	289 (238–386)	310 (244–354)	0.914

*Note*: Continuous variables are expressed as mean ± standard deviation or as median (interquartile range). Categorical variables are expressed as frequency (percent). Bold values are statistically significant.

Abbreviations: DYS, dyskinesia; HAMA, Hamilton Anxiety Scale; HRSD, Hamilton Rating Scale for Depression; H‐Y, Hoehn and Yahr stage; LEDD, levodopa equivalent daily dose; MMSE, Mini‐mental State Examination; MoCA, Montreal Cognitive Assessment; N1, the stage 1 of nonrapid eye movement sleep; N2, the stage 2 of nonrapid eye movement sleep; N3, the stage 3 of nonrapid eye movement sleep; NREM, non‐rapid eye movement; PD, Parkinson's disease; PSG, polysomnography; PSQI, Pittsburgh Sleep Quality Index; RBD, rapid‐eye movement disorders; REM, rapid eye movement; TST, total sleep time; UPDRS, Unified Parkinson Disease Rating Scale.

Compared with the non‐DYS patients, DYS patients had a lower proportion of male patients (*p* = 0.022), longer disease duration (*p* < 0.001), and higher LEDD (*p* < 0.001). In the assessment of motor symptoms, DYS patients had higher UPDRS IV scores (*p* < 0.001) and UPDRS II scores(*p* = 0.007). No significant differences in scores of PSQI, HAMA, HRSD, MMSE, and MoCA were found. There were no significant differences in UPDRS III scores between the two groups (Table 1).

### 3.2 Analysis of PSG data

Compared with non‐DYS patients, DYS patients had a significantly lower proportion of REM sleep percentage (*p* = 0.01) and REM sleep time (*p* = 0.006), higher proportion of NREM sleep (*p* = 0.006). There was no difference in other sleep parameters between the two groups (Table 1).

After adjusting for age, gender, age at onset of PD, LEDD, disease duration, taking amantadine or not, MoCA scores, NREM%, N3%, and REM sleep time (min), the percentage of NREM sleep (*p* = 0.035), female (*p* = 0.002), and LEDD (*p* = 0.005), and REM sleep time (min) (*p* = 0.012) were still associated with DYS, with OR = 0.91 (0.835–0.993), 0.051 (95% CI, 0.008–0.325), 1.006 (95% CI, 1.002–1.01), 0.965 (95% CI, 0.938–9.992) (Table 2).

**TABLE 2:** Binary logistic regression analysis of sleep parameters and dyskinesia in PD patients.

Variables	Single‐factor analysis			Multiple‐factor analysis		
	*β*	*p* value	OR (95% CI)	Standardized *β*	*p* value	OR (95% CI)
NREM, %	0.065	0.015	1.067 (1.012–1.125)	−0.794	**0.035**	0.91 (0.835–0.993)
Gender (female)	1.757	0.025	0.366 (0.152–0.879)	−2.951	**0.002**	0.051 (0.008–0.325)
Age, years	0.024	0.366	1.024 (0.972–1.079)	8.691	0.889	2.744 (0–3,891,549)
Age at onset, years	0.028	0.232	0.972 (0.929–1.018)	−8.526	0.896	0.389 (0–553,525)
LEDD, mg	0.004	0.000	1.005 (1.003–1.006)	1.845	**0.005**	1.006 (1.002–1.01)
Disease duration, month	0.021	0.000	0.317 (0.125–0.803)	−3.277	0.914	0.937 (0.288–3.05)
Amantadine	−1.149	0.015	0.205 (0.12–0.351)	0.201	0.846	1.205 (0.182–7.961)
MoCA	0.015	0.772	1.015 (0.92–1.125)	0.284	0.437	1.058 (0.917–1.221)
N3, %	0.018	0.302	0.982 (0.95–1.016)	0.182	0.588	1.013 (0.966–1.064)
REM, min	0.016	0.018	0.984 (0.972–0.997)	−1.328	**0.012**	0.965 (0.938–0.992)

*Note*: Bold values are statistically significant.

Abbreviations: LEDD, levodopa‐equivalent daily dose; MoCA, Montreal Cognitive Assessment; N3, the stage 3 of non‐rapid eye movement sleep; NREM, non‐rapid eye movement; PD, Parkinson's disease; REM, rapid eye movement; UPDRS, Unified Parkinson Disease Rating Scale.

### 3.3 EEG spectral analysis of PD patients with and without dyskinesia

A total of 85 patients in the first part whose data were available were divided into non‐DYS (*n* = 62) and DYS (*n* = 23) groups. Comparison of baseline clinical[A4] data and sleep structure between the two groups showed similar results (Table S1).

#### 3.3.1 Normalized average power spectral density of different frequency bands of different brain regions

The normalized average power spectral density of the whole brain region, frontal region, central region, and occipital region in the DYS and non‐DYS groups during the NREM period was calculated. During NREM sleep, there is no significant difference between the two groups of the normalized average power spectral density in different frequency bands (Table 3).

**TABLE 3:** Comparisons normalized averaged power spectral density of different frequency bands in different brain regions between the two groups.

Frequency band	DYS (*n* = 23)	Non‐DYS(*n* = 62)	*p* value
Whole brain			
SWA0.5–4 Hz	6.13 (5.39–6.92)	6.34 (5.9–6.85)	0.498
*θ* 4–8 Hz	1.04 (0.86–1.33)	1 (0.78–1.24)	0.440
*α* 8–13 Hz	0.33 (0.16–0.53)	0.35 (0.18–0.53)	0.782
*σ* 13–16 Hz	0.13 (0.06–0.25)	0.15 (0.78–0.25)	0.652
*β* 16–30 Hz	0.04 (0.01–0.07)	0.05 (0.02–0.08)	0.502
0.5–30 Hz	5.25 (3.96–7.18)	6.21 (4.47–8.66)	0.239
Frontal area			
SWA0.5–4 Hz	6.15 (5.68–6.95)	6.40 (6.11–1.1)	0.379
*θ* 4–8 Hz	1 (0.83–1.17)	0.94 (0.74–1.19)	0.285
*α* 8–13 Hz	0.33 (0.12–0.62)	0.35 (0.15–0.53)	0.805
*σ* 13–16 Hz	0.12 (0.05–0.26)	0.12 (0.07–0.22)	0.961
*β* 16–30 Hz	0.04 (0.01–0.07)	0.04 (0.02–0.07)	0.661
0.5–30 Hz	5.48 (4.8–7.27)	7.3 (5.3–9.71)	0.113
Central area			
SWA0.5–4 Hz	6.25 (5.62–6.87)	6.26 (5.88–6.8)	0.929
*θ* 4–8 Hz	1.08 (0.88–1.38)	1.00 (0.77–1.3)	0.340
*α* 8–13 Hz	0.37 (0.17–0.61)	0.35 (0.18–0.55)	0.972
*σ* 13–16 Hz	0.13 (0.06–0.26)	0.17 (0.08–0.27)	0.403
*β* 16–30 Hz	0.05 (0.01–0.07)	0.05 (0.02–0.08)	0.453
0.5– 30 Hz	4.95 (3.65–7.37)	5.97 (4.39–7.9)	0.437
Occipital area			
SWA0.5–4 Hz	6.42 (5.4–6.92)	6.21 (5.74–6.78)	0.890
*θ* 4–8 Hz	1.02 (0.88–1.35)	1.13 (0.86–1.37)	1.000
*α* 8–13 Hz	0.34 (0.16–0.52)	0.32 (0.19–0.51)	0.949
*σ* 13–16 Hz	0.13 (0.06–0.27)	0.16 (0.07–0.25)	0.569
*β* 16–30 Hz	0.04 (0.01–0.07)	0.05 (0.02–0.08)	0.403
0.5–30 Hz	5 (3.12–7.55)	4.4 (3.03–6.04)	0.476

*Note*: Bold values are statistically significant.

Abbreviations: DYS, dyskinesia; SWA, slow‐wave activity.

#### 3.3.2 Absolute average power spectral density and normalized average power spectral density of SWA (0.5–4 Hz, 0.5–2 Hz, and 2–4 Hz) during early and late sleep

**FIGURE 2:**
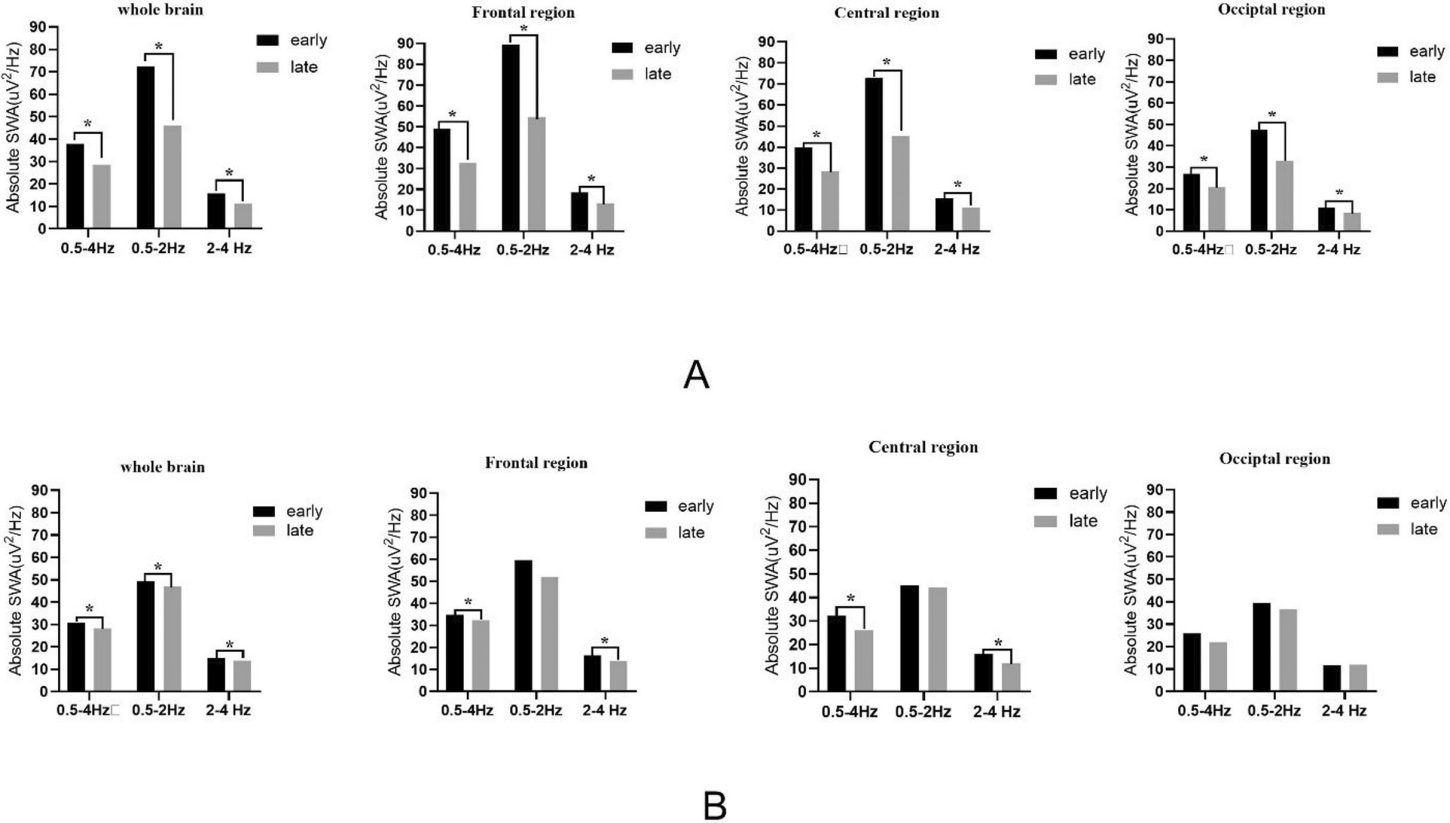
Changes in different bands of absolute average power spectral density of SWA during early and late sleep of different groups. Black rectangular “early” represents the absolute average power spectral density of SWA in early sleep, and the gray rectangular “late” represents the absolute SWA power spectral density of late sleep of PD patients. (A) Changes in absolute average power spectral density of SWA during early and late sleep of non‐DYS patients. (B) Changes in absolute average power spectral density of SWA during early and late sleep of DYS patients. The asterisks mean that there is a statistically significant difference between early and late sleep. DYS, dyskinesia; PD, Parkinson's disease; SWA, slow‐wave activity.

**FIGURE 3:**
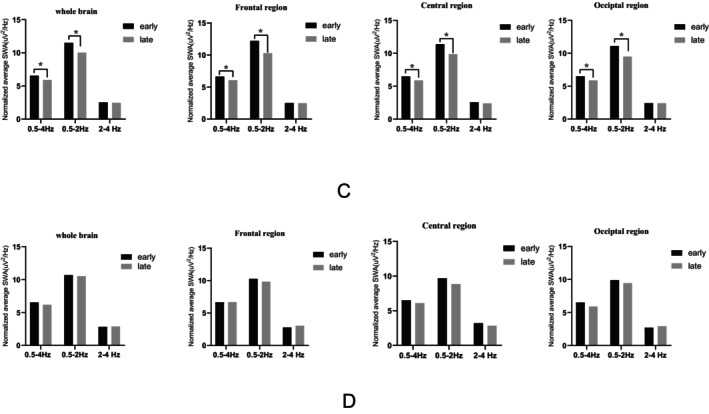
Changes in different bands of normalized average power spectral density of SWA during early and late sleep of different groups. Black rectangular “early” represents the normalized average power spectral density of SWA in early sleep, and the gray rectangular “late” represents the normalized average power spectral density SWA spectral density of late sleep. (C) Changes in normalized average power spectral density of SWA during early and late sleep of non‐DYS patients. (D) Changes in normalized average power spectral density of SWA during early and late sleep of DYS patients. The asterisks mean that there is a statistically significant difference between early and late sleep. DYS, dyskinesia; SWA, slow‐wave activity.

In NREM sleep of 62 patients in the non‐DYS group and 23 patients in the DYS group, the comparison of spectral density of SWA (absolute and normalized average power) in different brain regions in early and late sleep is shown in Figures 2 and 3.

The absolute power spectral density of whole brain region (*p* < 0.001), frontal region (*p* < 0.001), central region (*p* < 0.001), and occipital region (*p* < 0.001) SWA (0.5–2 Hz, 2–4 Hz, and 0.5–4 Hz) was significantly lower in late sleep than that of early sleep in non‐DYS group (Figure 2A).

In the DYS group, the absolute power spectral density of SWA (0.5–2 Hz, 2–4 Hz, and 0.5–4 Hz) of the whole brain region was significantly lower in late sleep than of early sleep (*p* = 0.015, *p* = 0.026, *p* = 0.002). The absolute power spectral density of frontal region SWA (0.5–4 Hz, 2–4 Hz) was significantly lower in late sleep than that of early sleep (*p* = 0.029, *p* = 0.007). The absolute power spectral density of central region SWA (0.5–4 Hz, 2–4 Hz) was significantly lower in late sleep than that of early sleep (*p* = 0.033, *p* = 0.003). There was no significant difference in the reduction of absolute power spectral density of any SWA frequency bands in the occipital brain region. There was no significant difference in the reduction of absolute power spectral density of SWA 0.5–2 Hz frequency bands in the frontal brain region of the 23 patients in the DYS group during early and late sleep (Figure 2B).

Changes in different bands of normalized average power spectral density SWA during early and late sleep in two groups are shown in Figure 3. In the non‐DYS group, compared with the normalized average power spectral density of SWA in late sleep, the whole brain region (*p* < 0.001), frontal region (*p* < 0.001), central region (*p* < 0.001), and occipital region (*p* < 0.001) normalized average power spectral density of SWA 0.5–4 Hz was significantly lower than that of early sleep. Similar changes were observed in the SWA 0.5–2 Hz (Figure 3C).

In the DYS group, there was no significant difference in the reduction of normalized average power spectral density of any SWA frequency bands in different brain regions (Figure 3D).

#### 3.3.3 Dynamic normalized average power spectral density of SWA (delta‐SWA) of DYS and non‐DYS group

On this basis, the normalized average power spectral density SWA (0.5–4 Hz, 0.5–2 Hz, 2–4 Hz) spectral density in early and late sleep of patients in the two groups was subtracted to obtain the normalized average power spectral density (ΔSWA (uV^2^/Hz)) of each patient. Compared with the non‐DYS group, the normalized average power spectral density of ΔSWA 0.5–4 Hz (0.19 vs. 0.6, *p* < 0.001) and normalized average power spectral density of low‐frequency ΔSWA 0.5–2 Hz (0.33 vs. 1.43, *p* < 0.001) were lower in the DYS group. Similar results were observed in different brain regions, including frontal, central, and occipital regions (Table 4).

**TABLE 4:** Normalized average power spectral density of NREM sleep ΔSWA (0.5–4 Hz, 0.5–2 Hz, 2–4 Hz) comparisons between the two groups.

ΔSWA	DYS (*n* = 23)	Non‐DYS (*n* = 62)	*p* value
Whole brain			
ΔSWA (0.5–4 Hz)	0.19 (−0.65 to 0.58)	0.6 (0.25 to 1.11)	**0.002**
Δlow‐SWA (0.5–2 Hz)	0.33 (−0.8 to 0.82)	1.43 (0.69 to 2.35)	**<0.001**
Δhigh‐SWA (2–4 Hz)	0.8 (−1.08 to 2.75)	0.19 (−0.05 to 0.61)	0.583
Frontal region			
ΔSWA (0.5–4 Hz)	0.01 (−0.83 to 0.18)	0.57 (0.24 to 1.01)	**<0.001**
Δlow‐SWA (0.5–2 Hz)	0.17 (0.33 to 0.92)	1.53 (0.84 to 2.36)	**<0.001**
Δhigh‐SWA (2–4 Hz)	0.06 (−0.4 to 0.35)	−0.02 (−0.21 to 0.2)	0.859
Central region			
ΔSWA (0.5–4 Hz)	0.08 (−0.53 to 0.32)	0.5 (0.17 to 1.02)	**0.003**
Δlow‐SWA (0.5–2 Hz)	0.29 (−0.88 to 1.51)	1.28 (0.71 to 2.36)	**0.011**
Δhigh‐SWA (2–4 Hz)	0.15 (−0.17 to 0.63)	0.01 (−0.2 to 0.2)	0.107
Occipital region			
ΔSWA (0.5–4 Hz)	0 (−0.97 to 0.28)	0.44 (0.15 to 1.04)	**0.001**
Δlow‐SWA (0.5–2 Hz)	−0.33 (−1.1 to 1.11)	1.3 (0.53 to 2.44)	**<0.001**
Δhigh‐SWA (2–4 Hz)	−0.17 (−0.38 to 0.24)	−0.08 (−0.29 to 0.14)	0.645

*Note*: Continuous variables are expressed as mean ± standard deviation or as median (interquartile range). Categorical variables are expressed as frequency (percent). Bold values are statistically significant.

Abbreviations: NREM, nonrapid eye movement; SWA, slow‐wave activity.

#### 3.3.4 Correlation between normalized average power spectral density of frontal ΔSWA and dyskinesia

Considering that DYS is a movement phenomenon, we selected frontal channels to analyze the correlation between the dynamic normalized average power spectral density of ΔSWA and DYS.

After adjusting the variables of gender, age, disease duration and LEDD, we found that only the normalized average power spectral density of ΔSWA 0.5–2 Hz in the frontal region (OR 0.657, 95% CI 0.46–0.939, *p* = 0.013) was independently correlated with the DYS (Table 5).

**TABLE 5:** Correlation between normalized average power spectral density of frontal ΔSWA (0.5–2 Hz) and dyskinesia.

	Single‐factor analysis			Multiple‐factor analysis		
	*β*	*p* value	OR (95%CI)	*β*	*p* value	OR (95%CI)
Frontal 0.5–2 Hz ΔSWA	−0.453	0.009	0.636 (0.453–0.892)	−0.456	**0.013**	0.657 (0.46–0.939)
Gender (female)	−0.722	0.145	0.49 (0.184–1.282)	0.402	0.605	1.495 (0.326–6.845)
Age, years	0.029	0.365	1.029 (0.967–1.095)	0.011	0.826	1.011 (0.917–1.114)
Disease duration, month	0.026	0.000	1.026 (1.013–1.04)	0.02	**0.056**	1.02 (0.999–1.041)
Amantadine	−0.885	0.094	0.413 (0.146–1.164)	2.315	0.059	10.13 (0.91–112.4)
LEDD, mg	0.004	0.000	1.004 (1.002–1.006)	0.002	0.125	1.002 (0.999–1.005)
REM latency, min	0.004	0.09	1.004 (0.999–1.008)	0.003	0.321	1.003 (0.997–1.01)
Sleep efficiency, %	−0.021	0.16	0.979 (0.95–1.008)	−0.032	0.294	0.968 (0.912–1.028)
REM, %	−0.054	0.077	0.95 (0.892–1.006)	−0.008	0.873	0.992 (0.898–1.095)

Abbreviations: CI, confidence interval; LEDD, levodopa‐equivalent daily dose; OR, odds ratio; REM, rapid eye movement; SWA, slow‐wave activity.

## 4 DISCUSSION

Our research shows that compared with non‐DYS PD patients, PD patients with DYS have changes in objective sleep structure. Specifically, the increased percentage of NREM sleep revealed by PSG indicates a greater risk of DYS in PD patients. The DYS group had lower frontal slow‐wave EEG average power spectral density during NREM throughout the night. Normalized average power spectral density of SWA 0.5–2 Hz reduction in the frontal region was still associated with DYS in logistic regression after adjusting for gender, disease duration, LEDD, age, and other confounding factors. We can regard it as a unique electrophysiological marker.

In humans, the decrease of SWA of EEG is related to the characteristic changes of several slow‐wave parameters, including the decrease in high‐amplitude waves, decrease of the slope of slow waves, and increase in the appearance of multi‐peak slow waves; all of which indicate the strength of cortical synaptic connections.^32^ All these indicate that many factors participate in slow‐wave sleep function rather than merely slow‐wave sleep ratio. EEG is a tool for studying the temporal dynamics of whole‐brain neuronal networks. We sought to investigate whether the average power spectral density of six channels in the whole brain region and the average power spectral density of two channels in each brain region were related to clinical indicators. The results of the average power spectral density of the whole brain represent the overall trend of brain power. It can be used as a basis for reference if further functional imaging studies are warranted.

DYS is related to changes in neural activity caused by impulsive stimulation of dopamine receptors in the basal ganglia. As a complex network, the corticothalamic system promotes the generation of various oscillation modes in a quiet sleep state, and these oscillations occur in the thalamus and cortex during NREM sleep.^33^ The damage to synaptic homeostasis during the disease process reaches a critical point, which exceeds the compensatory mechanism of cortical cortex and cortical synapse, resulting in loss of control of LTP related to DYS.

The relationship between motor symptoms and sleep is complex in PD patients. Meta‐analysis of the PSG in PD patients showed that the total sleep time, efficiency, slow‐wave sleep percentage, and the percentage of sleep from REM were significantly reduced in PD patients.^31^ Other factors could influence sleep architecture in PD patients. For example, with the increase in age, there will be an increase in sleep latency and awakening times.^34^ Some drugs can also affect the sleep structure, such as benzodiazepines, inhibit REM sleep, reduce the sleep time of slow‐wave sleep stage, and increase the sleep time of N2 stage. Excessive daytime sleepiness is a problem as many dopamine agonists are used for the treatment of PD. The mechanisms of sleep disorders in PD may be multifactorial. Potential influencing factors include degeneration of the central sleep regulation region, adverse effects of anti‐PD drugs, age‐related sleep changes, and the influence of motor symptoms.^35^ On the other hand, many studies revealed “sleep benefit” for motor symptoms in PD patients. The underlying mechanism between “sleep benefit” and the improvement of motor symptoms may be that sleep benefit was related to the relatively perfect reserve function of dopamine neurons.^36^ Higher accumulated power of sleep slow waves is associated with slower motor progression in PD patients.^37^ Poor nighttime sleep has been positively associated with DYS in our previous study.^10^ In DYS patients, there is a negative correlation between disease duration and the percentage of slow‐wave sleep.^17^ Our study found that DYS PD patients have higher percentage of NREM sleep and less REM sleep. Saccade velocity disturbance due to pathology of the brainstem itself, input to the brain stem or its output to premotor cortex may be recorded by PSG as decreased REM%.^38^ In PD, degeneration of brainstem nuclei impedes thalamocortical activation, resulting in disruption of REM sleep.^39^ While 5‐Hergic neurons were mainly concentrated in the raphe nucleus of the brainstem. Clozapine, the 5‐HT‐modulating compound has anti‐dyskinetic effect in PD indicating serotonergic system also participates in the occurrence of DYS in PD patients.^40^ All these may partly explain the significant reduction in the percentage of REM sleep alongside increased NREM in patients with DYS which was found in our study.

As mentioned previously, according to the synaptic homeostasis hypothesis,^41^ the regulation of intrinsic neural activity that occurs during sleep promotes the recovery of synaptic strength and maintains cellular homeostasis. This process is called LTP or long‐term depression (LTD). More preclinical and clinical research find the association between the increase of SWA and the synuclein burden.^15^ Among them, the index related to cortical synaptic strength is SWA, and the spectral density of SWA represents a reliable indicator of synaptic unit shrinkage during NREM sleep.^7^, ^8^

Galati et al. and Amato et al. measured the amplitude of synaptic decline during the entire sleep episode from electrophysiological and clinical aspects.^9^, ^17^ Their results show that impaired synaptic homeostasis and SWA NREM sleep‐induced reduction causes deterioration of LID. Our study provided the reduction of absolute and normalized average power spectral density of SWA during NREM sleep of two groups. Our study showed that compared to the non‐DYS group, there was no significant difference in the reduction of normalized average power spectral density SWA during NREM sleep in different brain regions of the 23 patients with DYS during early and late sleep (Figure 3D) which were consistent with Amato's findings.^17^ Among the sleep parameters, we only found that the percentage of NREM in the DYS group was higher, and it was positively correlated with DYS. The typical rhythms in NREM sleep include K‐complexes and SWA, which are important for the protection of sleep.^42^ Galati et al. observed that dyskinetic animals show an impairment of NREM sleep,^9^ particularly in SWA. Abnormal slow‐wave physiological activity, which in turn reduces the percentage of slow‐wave sleep, and the imbalance of circadian sleep homeostasis.

Amato et al. used high‐density EEG to find the correlation between DYS and sleep.^17^ In our study, six‐channel PSG technology was used to explore the correlation between sleep and DYS in a larger sample size. Further analysis of the EEG power spectral density showed that compared with patients without DYS, the average slow‐wave (0.5–4 Hz) EEG power spectral density of the frontal region was significantly lower in patients with DYS. Slow oscillations are the hallmark of deep sleep. Some measures such as rhythmic rocking, transcranial magnetic stimulation and sleep with no light can increase slow‐wave spectral density.^43^, ^44^, ^45^ As a result, it may enhance synchronous activity within thalamocortical networks, which in turn could promote sleep maintenance.^45^ Our study found that the average power slow‐wave (0.5–4 Hz) EEG spectral density in DYS patients was lower which is consistent with previous studies.^43^, ^44^, ^45^ This proves that patients without DYS have higher frequency slow‐wave oscillations and therefore have better deep sleep. It was confirmed that patients with DYS have subjectively poor sleep quality in our previous study.^10^ The current study also proves the conclusion objectively which is a further investigation of our previous study. However, we did not find the difference in slow‐wave sleep percentage between the two groups.

In our study, there was no significance of static SWA of whole‐night NREM sleep between the two groups. Whole‐night SWA/SWE can be considered dynamic SWA, often associated with cognitive impairment and motor progression.^16^, ^28^, ^37^ While early and late SWA is considered static SWA. the decline of early and late SWA has been linked with DYS^17^ and excessive daytime sleepiness.^46^ These parameters are likely to reflect different aspects of deep sleep. We also compared normalized average power spectral density and absolute SWA (early, late) within each group. The normalized average power spectral density of SWA in the whole brain region (*p* < 0.001), frontal region (*p* < 0.001), central region (*p* < 0.001), occipital region (*p* < 0.001), and the average power spectral density of SWA 0.5–4 Hz was significantly lower in late sleep than that of early sleep in patients without DYS but not in patients with DYS. After adjusting the confounding variables, the declining of frontal dynamic normalized average power spectral density of SWA 0.5–2 Hz is still associated with DYS indicating that impaired dynamic SWA regulation, especially in the frontal lobe may be associated with DYS in PD patients.

In clinical practice, we usually use medication to reduce the severity of DYS. Amantadine is widely used in clinical practice, but patients with moderate to severe DYS lack an effective curative effect after taking amantadine. Clozapine can significantly reduce the incidence and severity of DYS, but the potential risk of serious adverse events limits its use in clinical practice.^47^ In addition to drug treatment, noninvasive transcranial magnetic stimulation can improve the symptoms of DYS by stimulating the motor cortex. Although there is no research on whether the improvement of DYS after deep brain stimulation (DBS) is related to the regulation of NREM sleep, DBS is an effective adjunctive therapy for the management of motor symptoms, especially for PD patients with DYS. Several studies have found that subthalamic nucleus DBS (STN‐DBS) could also provide benefits for some nonmotor symptoms, notably sleep deregulation through normalization of sleep architecture. Patients who underwent DBS had greater delta (0–3 Hz) activity during NREM than during awake.^48^ Animal research about seizure revealed that DBS of the anterior nucleus of thalamus increased the amount of REM sleep, decreased the progressive enhancement of delta power during NREM sleep,^49^ and increased SWA, thereby increasing deep sleep and maintaining sleep continuity.^50^ All these studies help us understand how DBS improves NREM sleep, which is also beneficial to us for a better understanding of the role of NREM sleep and DYS pathogenesis. Schade et al. found that in N3 sleep, a specific auditory stimulus can amplify SWA in different frequency bands, which can more effectively stimulate low‐frequency power. These results indicate that some noninvasive neuromodulations which could improve sleep quality, especially NREM sleep could be used for alleviating DYS which is important for the quality of life in PD patients. With the development of an adaptive DBS system, EEG changes could be monitored and made adaptive adjustments which may provide more evidence for the relationship between nREM sleep and DYSs. It is still need to be proved that homeostatic SWA decline appears different between groups, but this remains to be proven and could be related to the changes in sleep architecture. Current technology may not be able to identify these biomarkers, but it can be done with more advanced analysis. Perhaps, techniques such as hypnodensity can contribute to helping us better understand sleep changes associated with movement disorders.^51^

Our study had some limitations. First, when using PSG to assess sleep quality, the patients could not avoid the first night effect.^52^ Secondly, this was a single‐center study, and there is a need to perform a larger, multi‐center study. Third, due to the small number of patients, the influence of specific drugs was not considered. In addition, although we took many measures to reduce the influence of body movement and sweating on the EEG recording, it is still impossible to avoid some artifacts which may affect the results. Furthermore, we could not establish a causal relationship between the occurrence of DYS and sleep due to observational study design. In addition, SWA was extracted from all epochs of NREM sleep will lead to artificially low SWA in a group with relatively high amounts of light NREM sleep. Also, we did not do multiple comparison corrections. In addition, linear mixed models, which may be more suitable for analyzing complex six‐channel EEG data. It will be considered in future studies.

In conclusion, our study provides evidence that some sleep and EEG parameters are promising markers of DYS in PD patients, and we verified the results of animal models and high‐density PSG clinical studies and confirmed the role of sleep, especially slow‐wave sleep in DYS in PD patients.

## AUTHOR CONTRIBUTIONS

Y.‐M.W., J.‐Y.L.J., data acquisition, statistical analysis, and writing of the draft. C.‐J.M., C.‐F.L., study concept and design. H‐Y.G., C.‐K.Z., C.J., L.K., J.H., X.‐Y.C., critical revision of the manuscript. Z.S., Y.‐L.G., J.‐R.Z., A.‐P.G., J.‐Y.L., C.‐J.M., C.‐F.L.X.X., revision of the manuscript.

## FUNDING INFORMATION

There is no financial disclosures. This study was financially supported by the National Natural Science Foundation of China (grant number 82071420, 82271279), Xiongan New Area Science and Technology Innovation Project(2023XAGG0073), Suzhou Key Laboratory of Sleep Disorders Diagnosis and Treatment Technology (SZS2023015), Gusu Health Talents Training Project (GSWS2019041, GSWS2020035), National Natural Science Foundation of China (82271279), Project of Jiangsu Provincial Health Commission (M2022063), and Project of Suzhou Provincial Health Commission (SKY2022048) Jiangsu Provincial Medical Key Discipline (ZDXK202217).

## CONFLICT OF INTEREST STATEMENT

No potential conflicts of interest relevant to this article were reported.

## Supporting information


Data S1.

## Data Availability

The data that support the findings of this study are available on request from the participants. Participants of this study did not agree for their data to be shared publicly, so supporting data is not available.
