# Supplementary material for: Lack variation of low slow‐wave activity over time in the frontal region in NREM sleep may be associated with dyskinesia in Parkinson's disease
Source: CNS Neurosci Ther. 2024 Oct 7;30(10):e70058. doi: 10.1111/cns.70058 (PMC11456717; doi:10.1111/cns.70058)
Supplement: Supplementary file 1 — Data S1. [file CNS-30-e70058-s001.docx]

**supplemental files**

clear

Fs=256; % Sampling frequency of the EEG data (256 Hz).

L=Fs*6; % Length of the window used for spectral analysis (calculated as 6 seconds of data at the given sampling rate).

T=1/Fs; % Sampling time interval (reciprocal of the sampling frequency).

t=(0:L-1)*T; % Time vector corresponding to the samples in the window.

Subj=[1:1];

[wn] = hann(L,'periodic'); % Window used for spectral analysis, initialized as a periodic Hanning window with the same length as the analysis window L.

for i=1:length(Subj)

% eeglab; % For all subjects

setname='_.set'; % File name

setpath=''; % File path

EEG = pop_loadset('filename',setname,'filepath',setpath); % For each subject specified in Subj, the code loads the EEG dataset using a function pop_loadset.

for ii=1:size(EEG.data,1)

for jj=1:size(EEG.data,3) % For each channel (jj) and trial (ii) in the EEG data.

y=squeeze(EEG.data(ii,:,jj));

[Pxx,F]= pwelch(y,wn,L*2/3,L,Fs); % The code performs a power spectral density (PSD) calculation using the pwelch function. The analysis window wn, overlap (L*2/3), and number of FFT points (L) are specified.

f_idx=find(F<=4&F>0.5);

p_final(ii,jj,:)=Pxx(f_idx); % Extraction of Frequency Bands：δ

f1_idx=find(F<=8&F>4);

p1_final(ii,jj,:)=Pxx(f1_idx); % Extraction of Frequency Bands：θ

f2_idx=find(F<=13&F>8);

p2_final(ii,jj,:)=Pxx(f2_idx); % Extraction of Frequency Bands：α

f3_idx=find(F<=16&F>13);

p3_final(ii,jj,:)=Pxx(f3_idx); % Extraction of Frequency Bands：σ

f4_idx=find(F<=30&F>16);

p4_final(ii,jj,:)=Pxx(f4_idx); % Extraction of Frequency Bands：β

f5_idx=find(F<=30&F>0.5);

p5_final(ii,jj,:)=Pxx(f5_idx); % Extraction of Frequency Bands：0.5-30Hz

end

end

end

a = mean(p_final); %The code calculates the mean power across trials and channels within each frequency band.

b = mean(a); %Takes the mean of a.
